# Opsoclonus‐Myoclonus‐Ataxia Revealing Underlying Neuromyelitis Optica Spectrum Disorder

**DOI:** 10.1002/mdc3.70823

**Published:** 2026-09-16

**Authors:** Ana Silvia Sobreira Lima Verde, Flávia de Paiva Santos Rolim, Amanda Vale Catunda, José Orlando Ferreira Tomé Filho, Milena Sales Pitombeira, Fernanda Martins Maia Carvalho, Paula Camila Alves de Assis Pereira Matos

**Affiliations:** ^1^ Movement Disorders Division, Department of Neurology Hospital Geral de Fortaleza Fortaleza Brazil; ^2^ Universidade de Fortaleza Fortaleza Brazil; ^3^ Neuroimmunology Division, Department of Neurology Hospital Geral de Fortaleza Fortaleza Brazil; ^4^ Center of Health Sciences Universidade Estadual do Ceará Fortaleza Brazil

**Keywords:** opsoclonus‐myoclonus‐ataxia syndrome, neuromyelitis optica spectrum disorder, AQP4‐IgG, movement disorders

A 24‐year‐old woman presented with a two‐month history of recurrent falls, diplopia, dysarthria, and irritability. At age 18 she had developed transient left‐leg hypoesthesia and gait unsteadiness, followed a year later by a brief peripheral facial palsy, and progressive cognitive decline over the subsequent 2 years.

On examination, she had bursts of back‐to‐back conjugate saccades without an intersaccadic interval and with a slight vertical component, characterizing opsoclonus (Video 1, Part A), with myoclonus, truncal and gait ataxia, binocular diplopia, reduced right‐eye visual acuity (20/50) with a right relative afferent pupillary defect, and mild dystonic posturing.

**Video 1:** Part A‐Neurological examination. Baseline assessment: opsoclonus, ataxic gait, trunk‐limb dyssynergia, dystonia. Part B‐Post‐immunotherapy: marked improvement in eye movements, gait, and dystonia.

MRI showed dorsal midbrain post‐contrast T1 enhancement at the inferior colliculi (Fig. 1(A), (B)), non‐expansile cervical cord lesions at C4–C6 (Fig. 1(C), (E)), and dorsal lesions from T4–T8. CSF showed no pleocytosis; ANA was positive at 1:640 (fine‐speckled) and anti‐SSA/SSB was high‐titer. Given the rarity of opsoclonus‐myoclonus‐ataxia syndrome (OMAS) in adults, extensive paraneoplastic, immunological, and infectious investigations were performed, including whole‐body CT, an intraneuronal antibody panel (anti‐Hu, –Yo, –CV2, −amphiphysin, −Ma1, −Ma2) by indirect immunofluorescence, serology (HIV, HTLV, syphilis), and CSF Gram stain, culture, and multiplex PCR; all were negative. Anti‐Argonaute antibodies, relevant to OMAS/NMOSD,^1^ were unavailable at our institution. Visual evoked potentials showed right optic pathway involvement.

**Figure 1:**
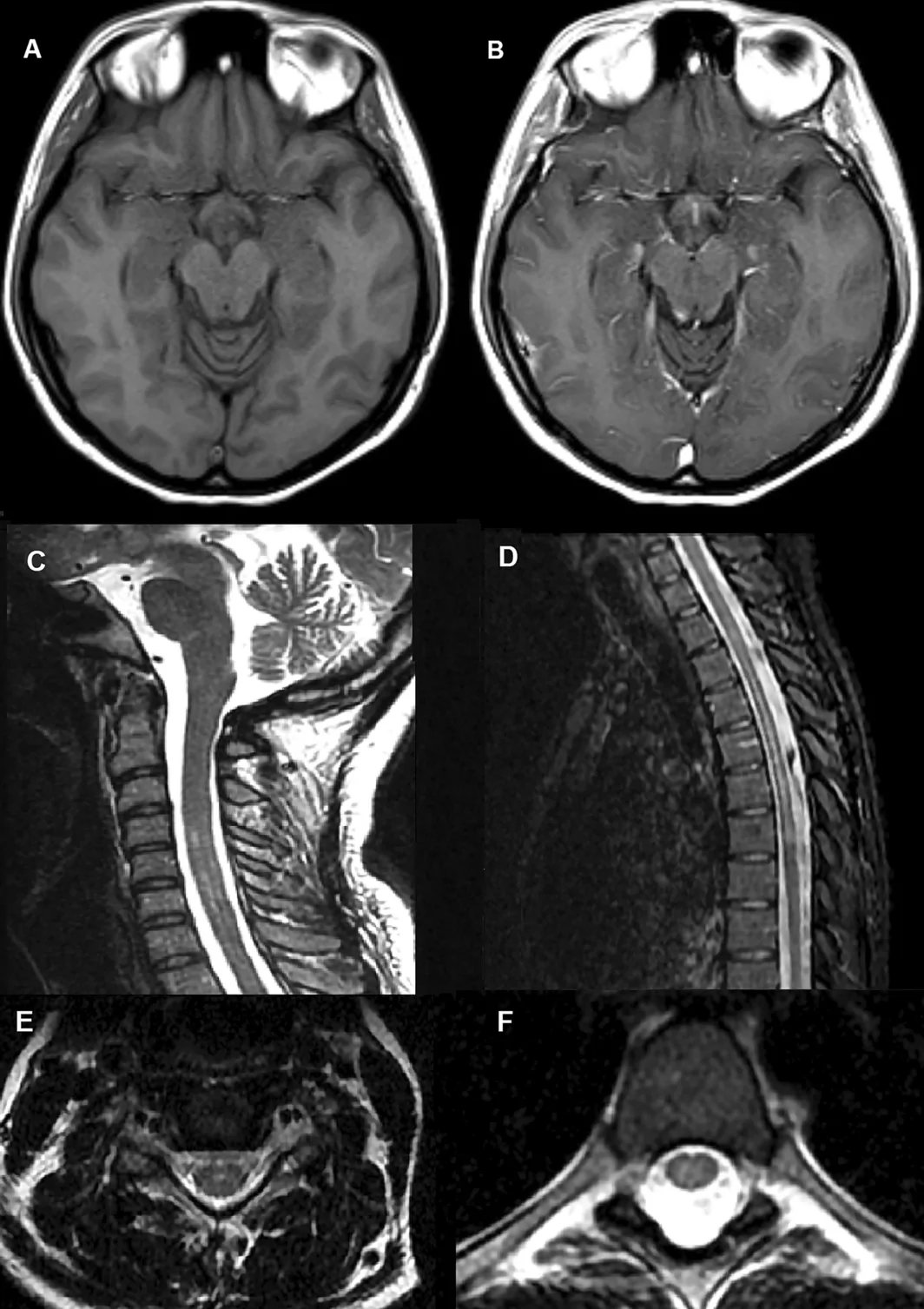
(A) Axial pre‐contrast T1‐weighted image showing no abnormality. (B) Post‐gadolinium axial T1‐weighted image demonstrating bilateral enhancement of the inferior colliculi. (C) Sagittal cervical T2‐weighted image showing hyperintense lesions C4–C6. (D) Sagittal thoracic T2‐weighted image demonstrating an extensive hyperintense lesion extending from T4 to T8. (E) Axial cervical T2‐weighted image showing hyperintense lesion at C5. (F) Axial thoracic T2‐weighted image showing hyperintense lesion at T5‐T6.

Neuro‐Sjögren syndrome was initially considered (ACR/EULAR score, 3 points) but ruled out after negative Schirmer test and minor salivary gland biopsy. Intravenous methylprednisolone (1 g/day for 5 days) produced partial improvement, and, given the initial suspicion of rheumatologic disease, six cycles of cyclophosphamide (750 mg per cycle) were administered. A serum live cell‐based assay detected high‐titer aquaporin‐4 IgG (AQP4‐IgG, 1:640), confirming neuromyelitis optica spectrum disorder (NMOSD) by the 2015 IPND criteria.^2^

In our public‐hospital setting, maintenance azathioprine (2.5 mg/kg/day) was initiated based on published evidence supporting its efficacy in NMOSD. At 15 weeks, the abnormal ocular movements had resolved and gait and dystonic posturing had improved, without serious adverse events (Video 1).

OMAS is defined clinically by opsoclonus with myoclonus and/or ataxia, often with behavioral and sleep disturbance. In adults it is usually paraneoplastic, parainfectious, or otherwise autoimmune and has no specific biomarker^3^; evaluation should exclude malignancy and infection and consider autoimmune encephalitis.

Opsoclonus/ocular flutter likely reflects dysfunction of the cerebellar‐brainstem saccadic burst/omnipause network and may occur without a clear MRI correlate.^3^ It has been reported in AQP4‐IgG‐positive NMOSD with brainstem involvement.^4^ Here, imaging was consistent with NMOSD but did not show involvement of structures typically linked to OMAS; an underlying microstructural lesion in cerebellar‐brainstem circuitry, undetectable on conventional MRI, remains plausible. Negative paraneoplastic and infectious studies helped exclude other common causes of OMAS.^3^

NMOSD diagnosis relies on AQP4‐IgG (preferably live cell‐based assay), core clinical features, MRI, and exclusion of mimics; AQP4‐IgG positivity with a single core event, including subclinical optic neuritis, is sufficient.^2^

Movement disorders in NMOSD include tonic spasms, dystonia, and ataxia; hyperkinetic phenotypes such as myoclonus are rare but do not exclude NMOSD, supporting AQP4‐IgG testing in atypical presentations.^5^

## Author Roles

(1) Case Report: A. Conception, B. Data Collection, C. Analysis, D. Execution; (2) Manuscript Preparation: A. Writing of the First Draft, B. Review and Editing.

A.S.S.L.V.:1A, 1B, 1C, 2A.

F.P.S.R.: 1A, 1B, 2A.

A.V.C.: 1A, 1B, 1C, 1D, 2A.

J.O.F.T.F.: 1A, 1B, 1C,1D, 2A.

M.S.P.: 1A, 1C, 1D, 2A.

F.M.M.C.: 1A, 2A.

P.C.A.A.P.M.: 1A, 1C, 1D, 2A, 2B.

## Disclosures

**Ethical Compliance Statement:** Institutional Review Board approval was not required for this clinical vignette. Written informed consent for publication of this case report, including clinical details, images, and video material, was obtained from the patient. We confirm that we have read the *Movement Disorders Clinical Practice* position on issues involved in ethical publication and affirm that this work is consistent with those guidelines.

**Financial Disclosures and Conflicts of Interest:** No specific funding was received for this work. The authors declare that there are no conflicts of interest relevant to this work. The authors declare that there are no additional disclosures to report. Author disclosures are available in the Supporting Information.

## Supporting information


**Data S1.** Supporting Information.

## Data Availability

The data that support the findings of this study are available on request from the corresponding author. The data are not publicly available due to privacy or ethical restrictions.
